# Spatiotemporal Profiling Defines the Epithelial and Mesenchymal Transition Window in Embryonic Lung Morphogenesis

**DOI:** 10.3390/jdb14020025

**Published:** 2026-06-01

**Authors:** Huiwen Zheng, Jinpei Lin, Hanyi Li, Shijie Hao, Mengnan Cheng

**Affiliations:** 1 College of Life Sciences, University of Chinese Academy of Sciences, Beijing 100049, China; zhenghuiwen5@gmail.com (H.Z.); linjinpei@genomics.cn (J.L.); 2 Key Laboratory of Spatial Omics of Zhejiang Province, BGI Research, Hangzhou 310030, China; 3 BGI Research, Shenzhen 518083, China; lihanyi@genomics.cn; 4 State Key Laboratory of Genome and Multi-omics Technologies, BGI Research, Hangzhou 310030, China

**Keywords:** epithelial–mesenchymal interaction, spatiotemporal atlas, GWAS meta-data, lung morphogenesis

## Abstract

Lung organogenesis is orchestrated by dynamic epithelial–mesenchymal interactions during embryogenesis, yet the gene regulatory programs and signaling dynamics governing these processes in the pseudoglandular stage remain incompletely understood. In this study, we integrated spatial and single-cell transcriptomic data across embryonic developmental stages to systematically characterize epithelial and mesenchymal dynamics during lung development. To achieve more refined cell types at single-cell resolution in spatial transcriptomic data, we developed a bin-based deconvolution strategy that enabled high-precision cell-type assignment. We subsequently constructed a 3D spatiotemporal landscape of lung development and elucidated the molecular regulatory mechanisms underlying epithelial–mesenchymal maturation during lung morphogenesis. In addition, we analyzed transcription factor module activity, intercellular communication signaling, and predicted downstream target genes, while integrating public GWAS metadata to link developmental programs with lung cancer-related features. We observed pronounced stage-specific functional heterogeneity between the pseudoglandular and late embryonic stages. Notably, E13.5 emerged as a critical transition window, during which progenitor states shifted toward more mature cellular phenotypes. We reconstructed epithelial–mesenchymal interactions and uncovered coordinated rewiring of ligand–receptor signaling and transcriptional networks across developmental stages. Regulatory network analysis further identified temporally coordinated transcription factor modules centered on *Tbx3*, *Tbx5*, *Gli1*, *Gata4/5*, *Foxa1/2*, and *Cebpa*, which collectively orchestrated branching morphogenesis, epithelial patterning, and tissue stabilization. Integration with lung cancer genome-wide association data demonstrated that embryonic lung progenitor states exhibit strong associations with lung cancer-related transcriptional programs, particularly involving epithelial–mesenchymal plasticity and RNA-splicing pathways. Furthermore, TP53/HNRNP-mutant lung adenocarcinomas displayed embryonic-like molecular features associated with cytoskeletal remodeling and progenitor-state reactivation. Together, our study provided a spatiotemporally resolved framework of embryonic lung development and identifies a critical transition window linking lung morphogenesis, regulatory network remodeling, and cancer-associated epithelial plasticity.

## 1. Introduction

Lung development in mice began with the emergence of lung buds from the ventral foregut around embryonic day 9.5 (E9.5) and reached full maturation approximately 1–2 weeks after birth [1,2]. This process progressed through a series of partially overlapping developmental stages, including the embryonic stage (E9.5–E12.5), pseudoglandular stage (E12.5–E16.5), canalicular stage (E16.5–E18.5), saccular stage (E18.5–birth), and alveolar stage (postnatal day 1–14) [3]. Each developmental stage was marked by discrete but smoothly coordinated transitions in cellular differentiation and tissue morphogenesis.

The earliest characterized events governed by Nkx2-1 during lung organogenesis were the formation of specialized respiratory domains and the emergence of initial lung primordia [4]. Lung bud formation and branching during the pseudoglandular stage depended on conserved signaling pathways, such as the Fgf10-Fgfr2 axis [5,6]. These events were orchestrated through epithelial–mesenchymal interactions that maintain a distal signaling center governed by pathways including BMP/TGF-β, WNT, SHH, and retinoic acid (RA) [3,7,8]. Notably, these pathways engaged in extensive cross-regulation: for instance, SHH signaling induces the expression of Wnt2 and Bmp4 in the mesenchyme, while Wnt7b/β-catenin signaling enhances Bmp4 and Fgfr2 expression in the epithelium to coordinate branching dynamics [9].

These signaling cascades cooperatively regulated downstream effectors within an intricate regulatory network. Among transcription factors, *Sox2* and *Sox9* established proximal–distal epithelial patterning, while FGF, WNT, and SHH signaling maintained Sox9+ distal tip progenitors essential for branching [10,11]. As pioneer transcription factors, *Foxa1* and *Foxa2* preserved chromatin competence in early respiratory endoderm [12,13], and at later stages, they further regulated epithelial differentiation and the expression of genes critical for lung function at birth. Analogous to *Foxa2* and *Nkx2-1*, *Gata6* was expressed in the airway epithelium during lung morphogenesis and required for the survival of endoderm-derived progenitors that give rise to the bronchiolar epithelium [1,14].

Beyond canonical pathways, the extracellular matrix (ECM) together with integrin-mediated adhesion provided essential structural and mechanical cues [15,16]. Notably, lung cancer hijacked this same suite of developmental programs—WNT, FGF, SHH, NOTCH, Hippo/YAP, ECM remodeling, and EMT—to induce pathological reactivation of progenitor proliferation, plasticity, and migration; these cellular behaviors were typically quiescent once lung tissue reached maturity [17,18,19].

Recent advances in spatial transcriptomics and single-cell multi-omics provided unprecedented opportunities to resolve developmental processes with spatial and temporal precision. In this study, we integrated spatial transcriptomic and single-cell RNA sequencing datasets spanning mouse embryonic lung development from E10.5 to E18.5 and developed a bin-based spatial deconvolution strategy enabling single-cell-resolved spatial annotation. Through this framework, we identified E13.5 as a critical developmental transition window characterized by coordinated epithelial and mesenchymal maturation, extensive remodeling of signaling interactions, and stage-specific transcription factor regulatory programs. Furthermore, integration with lung cancer GWAS data revealed developmental programs associated with epithelial plasticity and tumor-related transcriptional states.

## 2. Materials and Methods

### 2.1. Data Source

Spatial transcriptome data can be downloaded from: https://db.cngb.org/stomics/mosta/ (accessed on 25 November 2025).

Lung tissues were first identifiable in samples collected at E12.5. Samples at E13.5 were excluded from the results due to the absence of ssDNA (single-strand DNA) images. The size of the lung tissue dataset after extraction was 60 megabytes (60 M).

Single-cell data can be downloaded from: https://omg.gs.washington.edu/ (accessed on 1 December 2025).

Cells annotated as Lung_and_airway for major_trajectory were extracted from the metadata of the single-cell dataset and subjected to the analyses in this study. The size of the extracted data was 178 M, which comprised 260,000 cells.

### 2.2. Cell Segmentation and Preprocess

We performed spatial cell segmentation by integrating the nuclear staining image and the CID-tagged expression matrix from the same tissue section using StereoCell (https://github.com/BGIResearch/StereoCell (accessed on 28 November 2025)). The workflow consisted of three major steps. (1) Image-matrix registration: the staining image was aligned to the corresponding expression matrix to ensure spatial consistency. (2) Cell morphology segmentation: the staining image was first denoised using median filtering, followed by cell boundary detection with an enhanced U-Net deep learning model. The predicted masks were further refined through area filtering and morphological “erosion-dilation” operations to optimize cell shapes and boundaries. (3) UMI assignment and cell matrix construction: after identifying nuclear masks, UMIs located within nuclei were directly assigned to the corresponding cells. The remaining UMIs were probabilistically assigned using a Gaussian Mixture Model, where high-confidence assignments were used to recover cytoplasmic UMIs. Finally, UMIs belonging to the same cell were aggregated to generate the cell-by-gene count matrix for downstream analyses.

### 2.3. Cell-Type Annotation

(1) Data preprocess

Single-cell spatial transcriptomic data were aggregated into bin50-resolution data, and a correspondence map between each bin50 ID and its constituent cellbin IDs was generated. For the merged bin50 dataset, we constructed a counts matrix and a spatial coordinate matrix formatted according to the input requirements of CytoSpace. The reference dataset was processed in parallel to generate a counts expression matrix and a corresponding list of cell types.

(2) CytoSpace process

Spatial assignment of single-cell transcriptomes was performed using CytoSpace (v1.0.0) [20]. The method integrates scRNA-seq profiles with spatial transcriptomic cellbin data by optimizing a graph-based linear assignment model that matches cells to spatial locations based on transcriptional similarity under spatial constraints. Normalized scRNA-seq data and spatial expression matrices were supplied as inputs after restricting both datasets to shared variable genes. CytoSpace was executed using default parameters, and the resulting assignment matrix was used to determine the most probable spatial position for each cell. These inferred coordinates were subsequently used to reconstruct cell-type-resolved spatial distributions.

(3) Reference-based mapping of spatial transcriptomic bins using optimal transport

To assign spatial transcriptomic bins (bin50) to reference single-cell types, we developed a mapping framework based on optimal transport (OT) [21]. This framework leverages highly variable genes (HVGs), normalization strategies, and entropic regularization to achieve robust probabilistic assignments.

1. Preprocessing and feature selection:

Reference single-cell data and target cellbin data were jointly preprocessed to ensure comparability. Counts were first normalized to a total of 10^4^ per cell, followed by log-transformation and z-score scaling across genes. Highly variable genes (HVGs) were selected using the Seurat v3 method [22], and only the intersection of HVGs between datasets was retained. For computational efficiency, we subsampled at most 500 reference cells per annotated type.

Let ${X\in R}^{n\times g}$ denote the normalized expression matrix for *n* cellbins across *g* genes, and ${Y\in R}^{m\times g}$ the corresponding reference matrix with m cells.

2. Cost function:

We defined the cost between cellbin *i* and reference cell j as the correlation-based distance:

$$C_{ij}=1-coor\left(X_{i},Y_{j}\right)$$

where $X_{i},Y_{j}{\in R}^{g}$ are gene expression vectors. This metric captured dissimilarity while being robust to scaling.

3. Optimal transport formulation:

We then solved the optimal transport problem between source distribution ${a\in \Delta }^{n}$ (uniform mass across nn cellbins) and target distribution ${b\in \Delta }^{m}$ (uniform or prior-weighted mass across reference cells). The classical linear OT problem is formulated as:

$$\underset{T\geq 0}{\min}\left\langle T,C\right\rangle s.t.{T1}_{m}=a,T^{⊺}1_{n}=b,$$

where ${T\in R}^{n\times m}$ is the transport plan, C is the cost matrix, and 1 is a vector of ones.

In practice, we employed either the exact Earth Mover’s Distance (EMD) solver or entropic regularization (Sinkhorn algorithm) with regularization parameter λ:

$$\underset{T\geq 0}{\min}\left\langle T,C\right\rangle +\frac{1}{\lambda }\sum_{i,j}T_{i,j}\log T_{i,j}$$

The entropic term improves numerical stability and computational scalability.

4. Aggregation to cell types:

For each source cellbin, the transport mass assigned to reference cells was aggregated by annotated reference cell types. This produced a type probability distribution per cellbin:

$$pi(t)=\frac{\sum_{j\in I_{t}}T_{i,j}}{\sum_{i=1}^{m}T_{i,j}}$$

where $I_{t}$ denotes the set of reference cells of type t. The most likely type was assigned as the best match, and the associated probability score reflected the confidence of the mapping.

5. Integration with spatial bins:

At the bin50 level, all cellbins contained within a bin were processed jointly. If prior information about expected cell-type composition within a bin was available, it was incorporated into the target distribution b to guide transport. Otherwise, a uniform prior was used. Finally, all the lists of cellbins assigned the best match to the metadata of cellbin.h5ad were added.

### 2.4. Single-Cell RNA-Seq Preprocessing, Dimensionality Reduction, Clustering, and Differential Expression with Scanpy

Single-cell transcriptomic data were processed using Scanpy (v1.9.3). We filtered out cells with less than 500 expressed genes and less than 1000 total counts, and filtered out genes expressed in less than 20 cells. Gene expression values were normalized to a total count of 1 × 10^4^ per cell and log-transformed using sc.pp.normalize_total and sc.pp.log1p. Highly variable genes (HVGs) were identified across cells with sc.pp.highly_variable_genes under the “seurat_v3” selection method, and only HVGs were retained for downstream analyses. We followed the cell annotations from the reference. Lung cells (Eln^+^), a cell-type of lung epithelial origin at the late embryonic stage, were identified in the reference dataset.

The expression matrix was scaled to unit variance, and principal component analysis (PCA) was performed using sc.tl.pca. Neighborhood graphs were constructed using the top principal components (sc.pp.neighbors), followed by nonlinear dimensionality reduction via UMAP (sc.tl.umap). Cell clustering was performed using the Leiden community detection algorithm (sc.tl.leiden) with a resolution parameter optimized based on cluster stability and biological interpretability.

Marker genes for each cluster were identified through differential expression testing using sc.tl.rank_genes_groups with the Wilcoxon rank-sum test. Genes with an adjusted *p*-value < 0.05 and positive log fold change were considered significant markers. Cluster identities were assigned based on canonical marker genes and previous literature.

### 2.5. Cell–Cell Communication Analysis with CellChat

Cell–cell communication analysis was performed using the CellChat R package (v1.6.1) [23]. A CellChat object was first constructed using the normalized gene expression matrix derived from the scRNA-seq dataset. Overexpressed ligands, receptors, and interaction pairs were identified by applying the identifyOverExpressedGenes and identifyOverExpressedInteractions functions; we used the default parameters, after which the interactions were mapped onto a reference protein–protein interaction network. Communication probabilities between cell groups were inferred using a permutation-based framework, and the resulting interaction networks were aggregated to obtain pathway-level communication patterns. Intermediate CellChat objects were saved at each processing step.

For comparisons between biological conditions, CellChat objects corresponding to the distinct groups were integrated using the mergeCellChat function. Differential interaction analysis was conducted by re-estimating overexpressed ligands/receptors and extracting regulated signaling events through the subsetCommunication function, with the cutoff for differential expression set at log2 fold change (log2FC) > 0.5.

To visualize global communication changes, we generated normalized difference heatmaps using ComplexHeatmap, where positive values represent strengthened interactions and negative values represent reduced interactions. Row- and column-wise barplot annotations were included to summarize overall signaling gains or losses across pathways and cell types.

### 2.6. Ligand–Target Prediction Using NicheNet

Ligand–target regulatory inference was implemented using the NicheNet R package (v2.2.0) [24]. Prior to analysis, the required regulatory datasets—including the ligand–receptor interaction network, ligand–target weight matrix, and transcription factor–target gene network—were obtained from the NicheNet public repository (Zenodo, DOI: https://zenodo.org/record/7074291).

Within each sender cell population, differentially expressed genes were identified and filtered to retain putative ligands (log2FC > 0.5; adjusted *p*-value < 0.05). Ligand activity scores were computed using the predict_ligand_activities function, which evaluates the ability of each candidate ligand to predict the transcriptional response observed in receiver cells. Subsequently, ligand–target associations were derived using the get_weighted_ligand_target_links function based on the NicheNet prior model. Potential downstream targets were further selected for visualization using the prepare_ligand_target_visualization function, applying a ligand–target weight threshold of 0.5.

### 2.7. Regulatory Network Inference and Regulon Activity Analysis with pySCENIC

Transcriptional regulatory networks were reconstructed using the pySCENIC workflow (v0.12.1) [25]. Briefly, gene co-expression modules were first identified from the log-normalized scRNA-seq expression matrix using the GRNBoost2 algorithm [26]. Candidate regulons were then inferred by integrating cis-regulatory motif enrichment information from the hg38/mm10 motif database, yielding high-confidence transcription factor–target gene relationships. Subsequently, regulon activity scores were quantified in each cell using AUCell, producing a cell-by-regulon activity matrix.

To compare transcription factor activity across developmental stages and cell populations, AUCell scores were z-standardized and visualized using heatmaps. Regulons exhibiting stage-specific activation or lineage-restricted patterns were identified based on differential activity testing. All pySCENIC analyses were performed using default parameters unless otherwise specified.

### 2.8. GO Enrichment Analysis

Genes belonging to each transcription factor (TF) module were subjected to functional enrichment analysis using the MetaSpace platform (https://metascape.org/gp/index.html (accessed on 8 January 2026)). Enrichment results, including Gene Ontology (GO) and pathway terms, were exported as tabular files and subsequently imported into R for downstream visualization. All plots were generated using custom R scripts employing standard data-processing and plotting packages (ggplot2 v3.4.0) [27].

### 2.9. Genetically Informed Spatial Mapping

Genetically informed spatial mapping (gsMap) was used to identify spatially resolved cell populations associated with complex traits by integrating GWAS summary statistics with spatial transcriptomic data. gsMap models spatially resolved gene expression profiles to compute gene specificity scores (GSSs) for each spatial unit, reflecting cell-type or state-specific transcriptional activity. These GSS values are linked to genetic variants by assigning SNPs to genes based on transcription start and end site proximity and curated SNP-to-gene regulatory annotations. Trait heritability enrichment associated with each spatial unit is then estimated using stratified linkage disequilibrium score regression (S-LDSC). Statistical significance across spatial units within defined anatomical or developmental regions is subsequently aggregated using a Cauchy combination test, enabling high-resolution mapping of trait–cell associations across tissues.

### 2.10. Genomic Alteration Analysis of TP53-Mutant LUAD

Genomic alteration analysis was performed using the cBioPortal for Cancer Genomics platform (https://www.cbioportal.org/ (accessed on 21 February 2026)) based on the Lung Adenocarcinoma dataset (https://gdc.cancer.gov/about-data/publications/pancanatlas (accessed on 21 February 2026)). TP53-mutant LUAD samples were stratified according to the mutational status of HNRNP family genes into the HNRNP group and TP53 group. Comparative Genomic Alterations analysis was subsequently conducted using the cBioPortal web interface to identify significantly enriched co-altered genes between groups. The resulting alteration matrix was exported and further visualized in R (version 4.3.0) using custom scripts and the ggplot2 package.

## 3. Results

### 3.1. Spatial Transcriptomic Cell-Type Annotation

**Figure 1:**
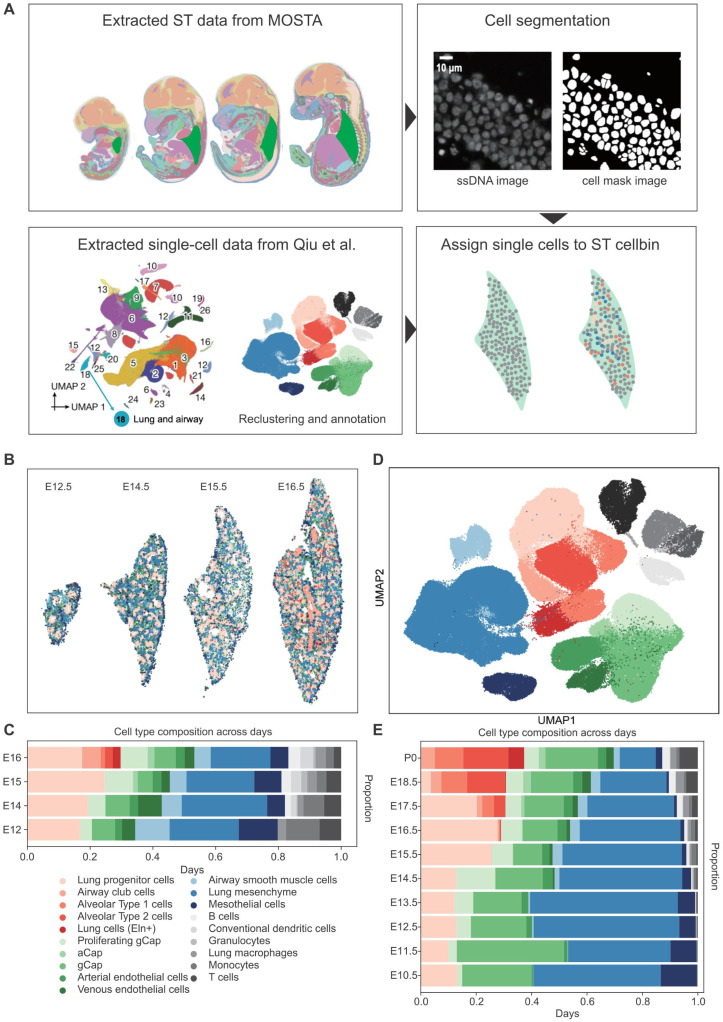
Schematic diagram and data overview of the study. (**A**) Spatial transcriptomic (ST) data from MOSTA: lung tissue regions extracted and cell segmentation performed. Single-cell data from Qiu et al. [29]: lung tissue-derived cells extracted, followed by reclustering and reannotation. Both ST and single-cell data were used for cell-type annotation. (**B**) Spatial visualization of cell-type annotations in embryonic lung spatial transcriptomic data. (**C**) Distribution of cell-type proportions across four developmental time points. (**D**) UMAP visualization of all lung-derived cell types identified from the reference dataset, colored by cell types. (**E**) Proportional distribution of the cell types shown in (**D**) across sequential developmental time points.

To investigate an epithelial and mesenchymal transition window in embryonic lung morphogenesis, we collected lung tissue data from mouse embryonic studies using a multi-time-point spatial transcriptome (ST) and single-cell RNA-seq [28,29]. ST data was downloaded from the MOSTA (Mouse Organogenesis Spatiotemporal Transcriptomic Atlas) website. To enable accurate cell-type annotation in ST data at single-cell resolution, we developed a novel bin-based spatial deconvolution annotation method. This approach was built upon CytoSpace [20], a previously reported deconvolution framework that supported ST data with variable spatial resolutions (Figure 1A,B).

Lung-derived cell populations were re-annotated and refined cell subtypes. Within endothelial populations, we identified lung-specific Car4^+^ Gpihbp1^+^ endothelial cells and further resolved their heterogeneity into general capillary endothelial cells (gCap, Gpihbp1^+^ Kit^+^), proliferating gCap cells (Mki67^+^ Gpihbp1^+^), and alveolar capillary endothelial cells (aCap) characterized by high *Car4* expression (Figure S1C) [30,31]. Characteristic expression of *Etv5* in lung epithelial cells, *Tbx5* in mesenchyme-derived cells, *Scgb3a2* in club cells, and *Pparg* in lung-resident macrophages were consistently validated in both the single-cell and spatial transcriptomic datasets, with their spatial distributions clearly resolved [32,33,34,35]. The annotation data were subsequently used as references for spatial deconvolution (Figure S1D,E).

Given that CytoSpace supported the specification of the expected cell number per compartment, we first aggregated cell-level transcriptomic data into bin-based units and established a mapping relationship between individual cell units and their corresponding bins. Following the annotation of bin-resolution ST data, we further deconvolved each bin according to its constituent cell IDs. Then, our novel bin-based annotation method consisted of four steps (Figure S2A). Firstly, informative feature genes were selected to ensure sufficient discriminative power for spatial decomposition while minimizing noise. Secondly, a cost matrix was constructed by computing cosine similarity between the expression profiles of cells across datasets, capturing intercell relationships. Thirdly, an optimal transport framework was applied to establish interpretable and continuous mapping between datasets with different spatial resolutions, enabling single-cell-level spatial reconstruction [36]. Finally, cell-type-level aggregation integrated high-resolution spatial geometry with low-resolution annotations and corrects annotation biases introduced by CytoSpace.

Compared with the CytoSpace single-cell annotation, cell types annotated by our method displayed spatial patterns that better matched expected anatomical organization, and the spatial localization of cell types was more consistent with the distribution of corresponding marker genes. In contrast, the conventional CytoSpace cellbin annotations exhibited dispersed and biologically implausible spatial patterns (Figure S2B).

In summary, we employed a single-cell resolved spatial cell-typing approach to integrate single-cell and ST dataset spanning the E10.5 to E18.5 window of mouse embryonic lung development (Figure 1B and Figure S1A). ST data provided positional context for gene expression, whereas single-cell transcriptomic data compensated for the limited capture efficiency of spatial profiling by delivering high-depth transcriptomic information at the single-cell level.

### 3.2. Identifying the Temporal Transition Point of Epithelial and Mesenchymal Maturation

**Figure 2:**
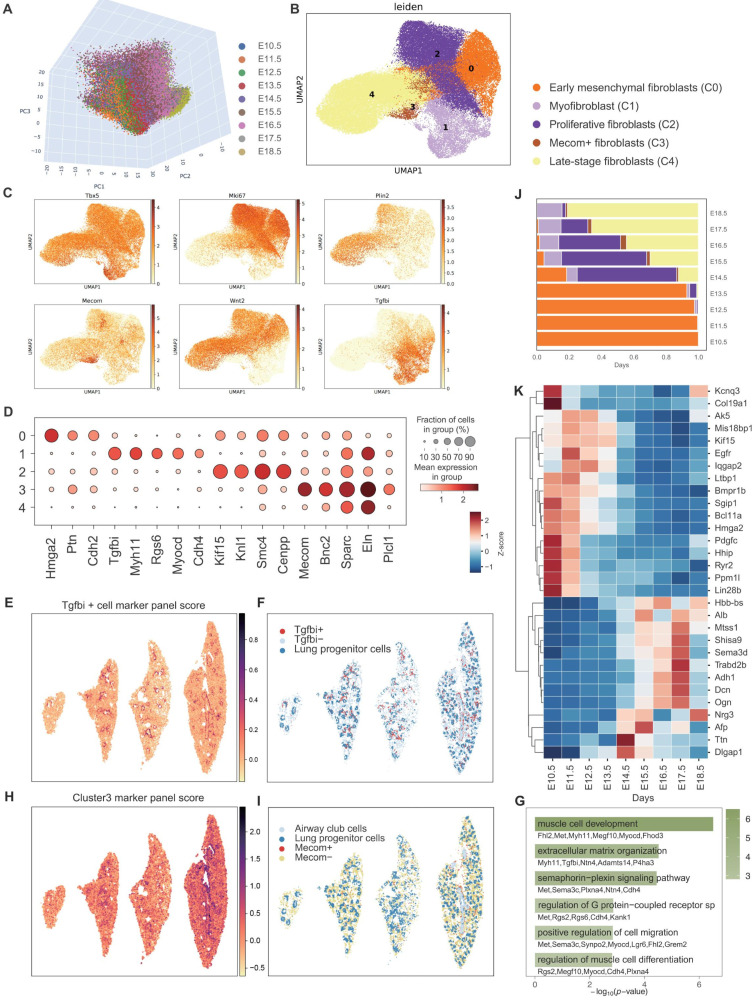
Identification of early and late mesenchymal signature genes and spatial localization of mesenchymal subtypes. (**A**) Principal component analysis (PCA) of mesenchymal cells, with each cell colored according to its developmental time-point origin, illustrating stage-dependent transcriptional divergence. (**B**) UMAP visualization of single-cell transcriptomic profiles, with cells colored by Leiden-derived clusters, defining major mesenchymal cell states. (**C**) UMAP visualization of signature gene expression across mesenchymal clusters. (**D**) Bubble plot visualization of signature genes in mesenchymal cell subtypes. (**E**) Spatial visualization of the Tgfbi^+^ subpopulation signature gene module score in 3D tissue space. (**F**) Cellbins with module scores > 0.2 in (**E**) were classified as the Tgfbi^+^ subpopulation; spatial distributions of Tgfbi^+^, Tgfbi^−^, and lung progenitor cells are shown. (**G**) Functional enrichment analysis of Cluster 1 signature genes. (**H**) Spatial visualization of the Cluster 3 signature gene module score. (**I**) Cellbins with module scores > 0.2 in (m) were classified as the Mecom^+^ subpopulation; spatial distributions of Mecom^+^, Mecom^−^, and lung progenitor cells are shown. (**J**) Proportional changes in the five mesenchymal subpopulations across sequential developmental stages. (**K**) Heatmap showing the expression patterns of early mesenchymal fibroblast signature genes across sequential developmental stages.

To identify the key transition point during which epithelial and mesenchymal populations shift from progenitor to late embryonic stages, we first performed principal component analysis (PCA) on all mesenchymal cells. 3D PCA representation clearly revealed the gradual transitions among time points within the spatial data (Figure 2A), while UMAP facilitated visualization of local transcriptional similarity and fine-scale cellular heterogeneity. Based on clustering and marker-gene signatures, we annotated five major mesenchymal subpopulations (Figure 2B). These subtypes included: early mesenchymal fibroblasts (C0), which highly expressed *Hmga2* and *Cdh2*; proliferative fibroblasts (C2) expressed *Wnt2* and *Mki67* similar to C0, indicating their active proliferative state; and late-stage fibroblasts (C4), which expressed *Plin2*, represented a continuous trajectory from early to late embryonic fibroblast stages [30]. The Tgfbi^+^ myofibroblast population (C1), characterized by *Tgfbi*, *Myh11*, and *Myocd* expression, localized spatially near epithelial progenitors (Figure 2E,F), suggested a potential role in airway wall construction [37]. A distinct Mecom^+^ population (C3) exhibited enriched expression of *Mecom* and localized to the bronchial region, surrounded by goblet cells (Figure 2H,I). Combined with functional enrichment analysis, C3 appeared to regulate processes such as cell migration and adhesion (Figure 2G), and might further contribute to bronchial smooth muscle cell formation as well as support goblet cell differentiation and secretory functions.

The proportion of early mesenchymal fibroblasts (C0) declined sharply from 93% to 18.5% after E13.5, accompanied by a marked increase in the proportions of C2 cells from 4.3% to 61.5% and C4 cells from 0.84% to 12.3% (Figure 2H). The gene expression pattern of C0 also underwent significant changes before and after E13.5. Specifically, genes enriched in E10.5-E13.5 C0 cells (*Kcnq3*, *Col19a1*, *Ak5*, *Mis18bp1*, *Kif15*, *Egfr*, *Lqgap2*, *Ltbp1*, *Bmpr1b*, *Sgip1*, *Bcl11a*, *Hmga2*, *Pdgfc*, *Hhip*, *Ryr2*, *Ppm1l*, *Lin28b*) were significantly downregulated after E13.5, whereas another gene set (*Alb*, *Mtss1*, *Shisa9*, *Sema3d*, *Trabd2b*, *Adh1*, *Dcn*, *Ogn*, *Nrg3*, *Ttn*, *Dlgap1*) was markedly upregulated (Figure 2I).

To characterize the progressive maturation of fibroblasts, we selected the temporally related subpopulations C0, C2, and C4, and performed differential expression analysis between early-stage cells (E10.5–E13.5) and late-stage cells at E18.5 (Figure S3A). Gene expression patterns displayed a clear gradient reflecting the decline of early-stage features and the establishment of mature programs (Supplementary Table S1). Early mesenchyme was enriched for cell cycle regulation, DNA replication, chromatin remodeling, and EMT, consistent with their high proliferative capacity and developmental plasticity (Figure S3B). *Lef1* and *Hmga2* suggested critical roles in early tube morphogenesis [38]. In contrast, late mesenchyme exhibited strong enrichment for ECM organization, muscle structure formation, lung morphogenesis, and vascular remodeling (Figure S3C). Upregulation of *Eln*, *Fn1*, *Fgf10* and *Tgfbr2* highlighted differentiation toward myofibroblasts, smooth muscle cells, and ECM-secreting fibroblasts that collectively supported airway and alveolar maturation [39,40].

Epithelial cells exhibited dynamics similar to mesenchymal cells (Figure S3D). Differential gene expression between early epithelium (E10.5–E13.5) and late epithelium (E18.5) revealed enrichment of early-stage programs associated with cell migration, cell–cell junction remodeling, and tube morphogenesis, consistent with ongoing airway branching (Figure S3E,F) (Supplementary Table S2). The expression of *Ntn1* and *Wnt7b* supported that epithelial cells remained in a phase of structural reorganization and polarity establishment, while enrichment of insulin-like growth factor (IGF) and metabolic pathways suggested heightened sensitivity to growth and metabolic cues [41]. Late-stage epithelial cells were enriched in surfactant homeostasis, lung development, endothelial migration, and small GTPase signaling (Figure S3G). The upregulation of *Sftpb*, *Abca3*, *Hopx*, and *Ager* reflected early differentiation toward alveolar type I (AT1) and type II (AT2) cell fates, and indicated engagement in alveolarization and epithelial–endothelial crosstalk [42].

### 3.3. Epithelial–Mesenchymal Crosstalk from Embryonic to Pseudoglandular Stages

**Figure 3:**
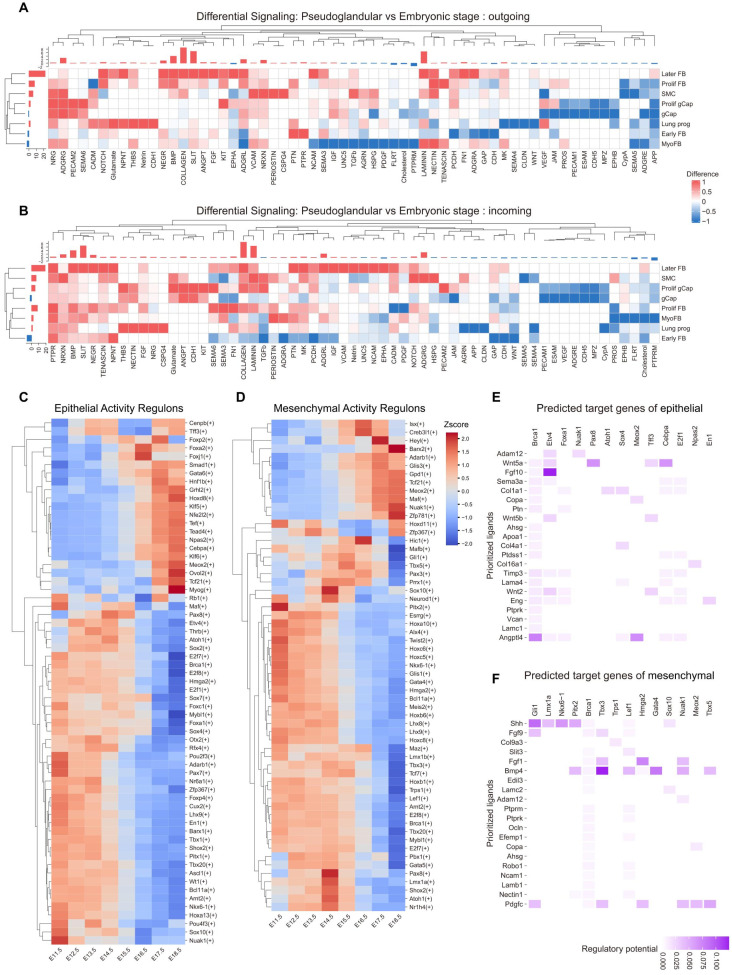
Signaling and TF activity changes in epithelial and mesenchymal cells. (**A**,**B**) Dynamics of outgoing (**A**) and incoming (**B**) cell–cell signaling activities from the embryonic to pseudoglandular stages. Positive values indicate increased signaling strength, whereas negative values indicate reduced activity. (**C**,**D**) Heatmaps displaying normalized transcription factor (TF) module activities in epithelial (**C**) and mesenchymal (**D**) cells across developmental time points. (**E**) Predicted association potential between epithelial signature transcription factors and ligands derived from mesenchymal cells, shown as a heatmap with mesenchymal cells as signal senders and epithelial cells as receivers. (**F**) Predicted association potential between mesenchymal signature transcription factors and ligands derived from epithelial cells, shown as a heatmap with epithelial cells as signal senders and mesenchymal cells as receivers.

During the transition from the early embryonic stage to the pseudoglandular stage of lung development, epithelial–mesenchymal signaling underwent extensive remodeling, reflecting the stage-specific reorganization of cell–cell communication necessary for branching morphogenesis [43]. Cell–cell communication analysis revealed that proliferating mesenchymal cells and late-stage mesenchymal cells shared similar outgoing signaling patterns. The outgoing intensity of several pathways-including laminin, ADGRL (Adhesion G Protein-Coupled Receptor L), NEGR (Neuronal Growth Regulator), BMP (Bone Morphogenetic Protein), collagen, Slit, and adhesion-associated pathways such as Nectin, Tenascin, Pcdh (Protocadherin), Fn1 (Fibronectin 1), and Adgra declined during the pseudoglandular stage (Figure 3A).

In terms of signal reception, both proliferating mesenchymal cells and late-stage mesenchymal cells show elevated input through BMP, Slit, NEGR, collagen, laminin, and ADGRL pathways, indicating that mesenchymal cells continuously sensed regulatory cues from the ECM and guidance molecules. CADM (Cell Adhesion Molecule), PDGF (Platelet-Derived Growth Factor), WNT, CDH (cadherin), and GAP junction pathways were markedly strengthened in late-stage mesenchymal cells while remaining low in proliferating mesenchymal cells (Figure 3B). The increased activation of CADM, PDGF, and cadherin signaling in late-stage mesenchymal cells indicates a functional shift from proliferative expansion toward mechanical stabilization of the airway structure, consistent with previous reports that PDGF signaling regulates fibroblast differentiation and matrix deposition.

During the pseudoglandular stage, FGF signaling became increasingly prominent. Our data also revealed stage-dependent changes in additional pathways, including decreasing input via AGRN (Agrin), APP (Amyloid Precursor Protein), and CLDN (claudin) and increasing input via THBS (Thrombospondin), Nectin, FGF (Fibroblast Growth Factor), NRG (Neuregulin), and CSPG4 (Chondroitin Sulfate Proteoglycan 4) (Figure 3B). This finding indicated that epithelial cells might enter a phase of active morphological shaping during this developmental window.

Additionally, we identified TF regulons in epithelial and mesenchymal populations and characterized their dynamics (Figure 3C,D). Before E14.5, both lineages exhibited a larger number of highly active TF regulons, whereas fewer regulons were upregulated at later stages. This pattern indicated that major lineage specification and axis patterning occurred in the early to proliferative state.

Next, we employed NicheNet analysis to predict the regulatory potential between prioritized ligands and downstream transcription factors (TFs). Based on mesenchyme to epithelium regulatory potential, we identified the Fgf10–Etv4 axis as a high-confidence regulatory node, consistent with its known role in orchestrating branching morphogenesis through FGF-ETS signaling [44]. Furthermore, WNT signaling was upregulated in late-stage lung epithelial cells, and *Wnt5a* was predicted to strongly regulate *Pax8* and *Cebpa*, indicating its involvement in epithelial lineage commitment in the late-stage of lung development. Interestingly, our analysis also highlighted several uncharacterized regulatory pairs, such as Angptl4–Meox2 and Col1a1–Atoh1, which exhibited high regulatory potential but remained largely unexplored in the context of pulmonary organogenesis.

A regulatory network centered on the Shh-Gli1/Tbx2/3 axis was shown in epithelium to mesenchyme regulatory potential, confirming the pivotal role of epithelial-derived Shh in orchestrating mesenchymal expansion and branching morphogenesis. We also observed strong potential for *Bmp4* and *Pdgfc* to modulate mesenchymal identity through TFs such as *Gata4* and *Hmga2*. Notably, we identified several unanticipated regulatory interactions, including the Slit3-Trps1 and Efemp1-Lef1 pairs.

### 3.4. Mesenchymal and Epithelium Patterning During Lung-Branching Morphogenesis

During the lung-branching stage, several regulons played important roles in the cell migration and maintenance of tissue integrity including *Tbx3*, *Tbx5*, *Gata4*, *Gata5* and *Gli1*. We characterized mesenchymal cells in the proliferative stage by the activation of the Tbx5(+) regulon. This regulon list included *Tbx3*, *Meis1*, *Zeb2*, and Rho GTPase-related genes (*Daam2*, *Arhgap42*, *Robo1*, *Slit3*), enabling cytoskeletal remodeling and directed migration (Figure S4F).

The activity of the Gli1(+) module gradually increased during lung morphogenesis. It served as a central regulatory hub integrating SHH, FGF, and PDGF networks (Figure 3F). The Gli1(+) regulon gene list included TFs (*Tbx3*, *Tbx5*, *Sox5*, *Hoxb3*, *Zeb2*), ECM, as well as adhesion molecules (*Col3a1*, *Col16a1*, *Vcan*, *Cdh11*, *Nrcam*, *Lama2*, *Sdc2*), and cytoskeletal regulators (*Daam2*, *Rnd3*, *Arhgap28*). This regulon synchronized ECM composition, signal gradient transmission, and directional migration to maintain tissue integrity during branch elongation (Figure S4E).

We also found that *Gata4* and *Gata5* were highly expressed in both epithelial and mesenchymal compartments, and notably, their regulatory module activities were markedly elevated in mesenchymal cells. We further identified a potential regulatory relationship between *Bmp4* and *Gata4* (Figure 3D). Previous studies had demonstrated that *Gata4* functioned as a downstream effector of BMP signaling in the lateral plate mesoderm and that conserved enhancer elements of *Gata4* contained FOX binding motifs [45]. Consistently, we observed co-expression of *Gata4/Gata5* and *Foxf1* in E11.5 embryonic lung tissue. *Gata4* controlled ECM synthesis (collagen family, *Mmp16*), WNT/Hedgehog interactions (*Sfrp1*, *Ptn*), and metabolic pathways (*Slc38a4*). *Gata5* enhanced Hedgehog/WNT integration (*Gli2/3*, *Wnt2*) and regulated ECM modification and migration genes (*Hpse2*, *Chst8*), promoting fibroblast and smooth-muscle differentiation and supporting epithelial patterning. The epithelial cells Gata6(+) regulon governed epithelial polarity (*Llgl2*, *Ocln*), adhesion (*Cdh8*, *Itga3*), and TGF-β/BMP pathways (*Tgfb2*, *Bmp4*) drove alveolar epithelial lineage specification and mesenchymal behavior (Figure S4H,I).

During early development, high activity of Gata4(+), Gata5(+), and Tbx3(+) regulons promoted ECM assembly (*Lama2*, *Col3a1*, *Sdc2*). ECM–integrin interactions with α3β1 and α6β1 (*Itga3*, *Itgb1*, *Itga6*) activated FAK-Src, PI3K/Akt, and MAPK/ERK pathways, enhancing survival, migration, and proliferation consistent with the dynamic remodeling state of early mesenchyme [46,47]. A shift in proliferative-stage mesenchymal cells from *Tbx3* to *Tbx5* was accompanied by strong upregulation of Rho GTPase regulators (*Robo1*, *Dennd2b*, *Arhgap42*), enabling cytoskeletal remodeling and directed migration. This corresponded to the transition from a highly plastic early mesenchyme to a more structurally organized pseudoglandular mesenchyme [48].

In epithelial cells, the Foxa1(+) regulon exhibited the highest activity in embryonic and pseudoglandular stages (Figure 3C). The gene module regulated by Foxa1 was enriched in epithelial branching, adhesion, differentiation, and EMT suppression (Figure S4A). The Etv4(+) regulon, acting downstream of FGF10 signaling, regulated genes involved in proliferation, migration, and epithelial polarity, including Id2, Dock5, and Unc5d. At later developmental stages, Foxa2 (+) and Cebpa (+) regulons became predominant (Figure 3C). Foxa2 was essential for epithelial lineage commitment and branching morphogenesis, and it contained differentiation TFs (*Sox9*, *Id2*, *Etv5*, *Elf5*) and multiple signaling mediators that ensured epithelial responsiveness (Figure S4A). Cebpa(+) regulon upregulation marked the initiation of epithelial maturation, consistent with CEBPA-mediated NKX2-1 recruitment during AT2 cell differentiation [49]. Its regulon included *Sftpb* and *Abca3*, indicating that the AT2 program was already established during the pseudoglandular stage (Figure S1G).

Collectively, these results defined a stage-resolved regulatory hierarchy, in which early Tbx3-driven programs were progressively replaced by Tbx5- and Gli1-dependent networks that coordinated cytoskeletal remodeling and ECM organization. While previous studies implicated Tbx5 in cardiopulmonary development [50], our results demonstrate that Tbx5 activation coincided specifically with the pseudoglandular stage and was linked to Rho GTPase-mediated cell migration, and highlighted a previously unappreciated temporal role in lung morphogenesis [51].

### 3.5. Pathway-Specific Cell–Cell Communication Networks in Embryonic Mouse Lung Revealed by CellChat

**Figure 4:**
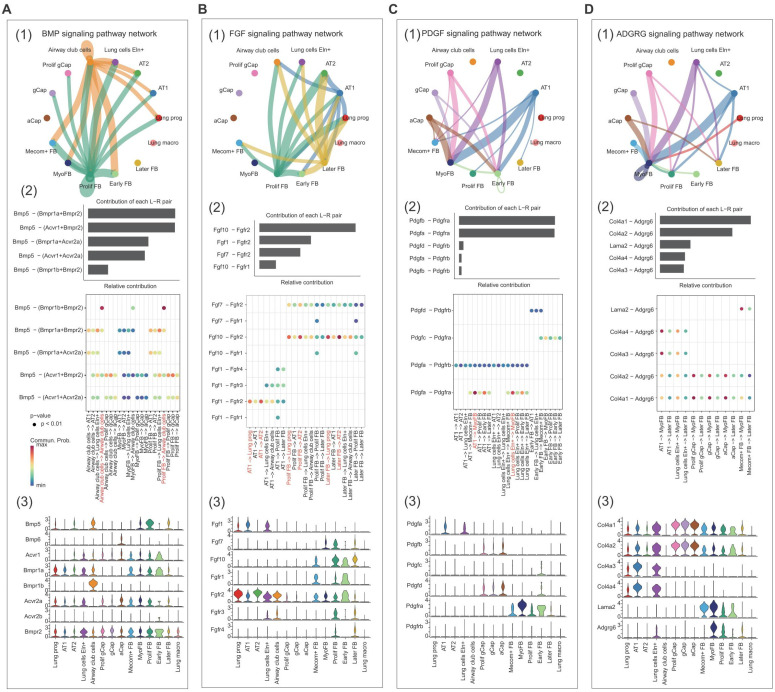
CellChat-based analysis of BMP, FGF, PDGF, and ADGRG signaling networks in embryonic mouse lungs. (**A**–**D**) Cell–cell communication analysis for BMP (**A**), FGF (**B**), PDGF (**C**), and ADGRG (**D**) signaling pathways. (**1**) Circle plots depict inferred signaling networks among lung epithelial, endothelial, mesenchymal, and immune cell-types, with edge thickness representing communication strength. (**2**) Bar plots show the relative contribution of major ligand–receptor pairs within each signaling pathway, and dot plots indicate the interaction strength between specific sender–receiver cell-type pairs (only interactions with *p* < 0.01 are shown). (**3**) Violin plots display the expression distribution of pathway-associated ligands and receptors across different cell populations.

To characterize intercellular communication in embryonic mouse lungs, we applied CellChat to infer ligand–receptor interactions among epithelial, endothelial, mesenchymal, and immune cell populations, focusing on BMP, FGF, PDGF (Platelet-Derived Growth Factor), and ADGRG (Adhesion G Protein-Coupled Receptor Subfamily G) signaling pathways (Figure 4A–D).

BMP signaling was dominated by mesenchymal fibroblast populations, particularly proliferative and airway club cell subsets, which primarily acted as signal senders, whereas epithelial lineages, including lung epithelial progenitors, AT1, AT2, and airway club cells, served as major receivers (Figure 4A). Multiple Bmp5-mediated ligand–receptor pairs, including Bmp5–(Bmpr1a + Bmpr2) and Bmp5–(Acvr1 + Bmpr2), accounted for most pathway activity. Correspondingly, Bmp5 was broadly expressed in fibroblasts, while BMP receptors were widely detected in epithelial and endothelial populations.

FGF signaling exhibited strong mesenchymal-to-epithelial communication (Figure 4B). Proliferative, embryonic late-stage fibroblasts and AT1 were the principal sources of FGF ligands, whereas lung epithelial progenitors and AT2 were major targets. Among the identified interactions, Fgf10–Fgfr2 contributed most prominently, followed by Fgf1–Fgfr2 and Fgf7–Fgfr2. Expression patterns showed enrichment of *Fgf10* and *Fgf7* in fibroblast populations and widespread expression of *Fgfr2* in epithelial cells.

PDGF signaling displayed a more restricted interaction pattern (Figure 4C), characterized by epithelial-derived ligands and mesenchymal-restricted receptors. Pdgfa–Pdgfra and Pdgfd–Pdgfra represented the dominant ligand–receptor pairs, with *Pdgfra* expression largely confined to fibroblast subsets.

In contrast, ADGRG signaling was primarily mediated by extracellular matrix-associated interactions (Figure 4D). Collagen ligands, including *Col4a1*, *Col4a2*, *Col4a3*, and *Col4a4*, interacted with Adgrg6 expressed in fibroblast populations, particularly proliferative and late-stage fibroblasts. Contribution analysis identified Col4a1–Adgrg6 and Col4a2–Adgrg6 as the major interactions, consistent with their corresponding gene expression patterns.

Collectively, these analyses reveal distinct, pathway-specific cell–cell communication architectures in the embryonic lung, highlighting diverse modes of epithelial–mesenchymal and matrix-associated signaling.

### 3.6. Developmental Suppression of Lung Cancer-Associated Traits of Epithelial Plasticity

**Figure 5:**
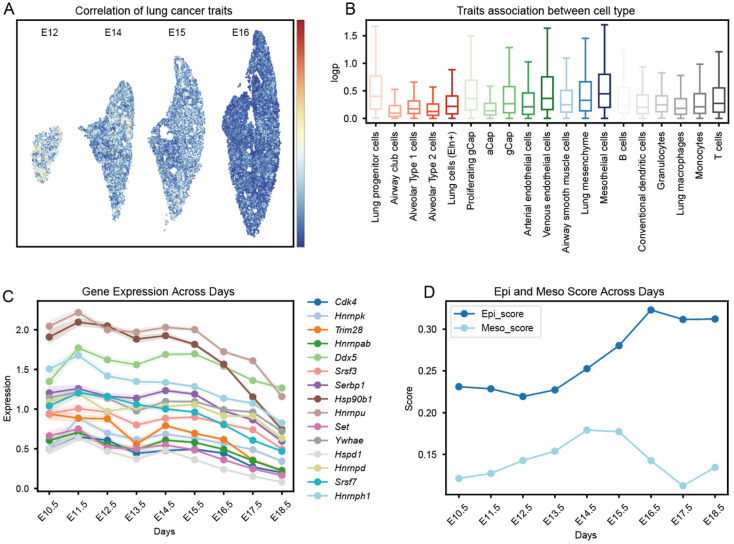
Spatiotemporal alterations of lung cancer-related traits. (**A**) GsMap spatial visualization results of the correlation of lung cancer characteristics in embryonic lung tissue. (**B**) The correlation between the significance of lung cancer traits and the types of embryonic lung cells. The X-axis shows different cell types, and the Y-axis shows the Pearson correlation coefficient. The box plot is colored by cell types. In each box, the median line represents the median, the gap represents the 95% confidence interval, the box represents the interquartile range, and the whiskers extend to 1.5 times the interquartile range. (**C**) Dynamic expression patterns of high-GSS genes in embryonic lung progenitor cells from E10.5 to E18.5. X-axis: Embryonic development stage (E10.5–E18.5); Y-axis: average value of gene expression. Lines of different colors represent different genes. (**D**) Dynamics of epithelial- and mesenchymal-state scores during embryonic lung development.

During embryonic development, organs exhibited tumor markers at specific stages [52]. Taking lung development as an example, lung cancer cells often performed states of distal epithelial progenitors in embryo development [53]. We integrated spatial transcriptomics data with summary statistics derived from genome-wide association studies (GWAS) to systematically map individual cell types or cell states to human complex traits, including a wide range of diseases using gsMap [54]. We extracted GWAS summary statistics from a former study [55] (https://opengwas.io/datasets/ukb-b-14521 (accessed on 2 February 2026)), and found that lung cancer traits showed a higher correlation in the earlier stage of lung development, and represented a progressive decline afterwards (Figure 5A and Figure S5A). Lung progenitor cells, venous endothelial cells, lung mesenchyme and mesothelial cells exhibited stronger trait association (Figure 5B). Further examination of high-GSS genes within lung progenitor cells revealed marked enrichment specifically in mRNA splicing, mRNA metabolic regulation, and the EMT pathway (Figure S5B).

To include more time points, we collected single-cell data of E10.5–E18.5, and calculated GSSs (gene specificity scores) for lung cancer traits. The expression of cancer cell markers consistently decreased during development (Figure 5C). We next quantified lung progenitor cell states during lung development, based on expression of E-cadherin, ZO-1, claudins, occludin, cytokeratins, and type IV collagen for epithelial scores, and vimentin, fibronectin, N-cadherin, and type I collagens for mesenchymal scores [56] (Figure 5D and Figure S5D). The epithelial score remained relatively stable in E10.5 to E13.5, and then rose until E16.5. The peaks of mesenchymal scores were at E14.5 and E15.5, indicating that by E14.5–E15.5 the lung resided in a late-branching phase with persistent epithelial–mesenchymal hybrid states (Figure 5D).

High-GSS genes were highly enriched for heterogeneous nuclear ribonucleoprotein (HNRNP) family members and RNA splicing regulators. Previous studies have reported that elevated HNRNPU protein expression is associated with poor overall survival in non-small cell lung cancer (NSCLC), supporting a tumor-promoting role for HNRNP-mediated RNA regulation. We stratified 265 TP53-mutant LUAD patients according to the presence of HNRNP family mutations, resulting in a TP53-mutant group of 203 patients (TP53 mutants but not HNRNP mutants) and an HNRNP-mutant group of 62 patients (both TP53 and HNRNP mutants). Genomic alteration analysis demonstrated that the HNRNP group exhibited a distinct co-alteration landscape enriched for genes including CTNNA3, ENAH, CDC42BPA, MYCN, LEFTY2, MIB1, ZBED2, HK1, and OSBPL1A (Figure S5E).

Several altered genes enriched in the HNRNP group were closely associated with embryonic developmental programs. LEFTY2, a TGF-β superfamily member involved in embryonic patterning and stem-cell regulation, and MYCN, a developmental oncogene-controlling progenitor proliferation and lineage specification, suggested partial reactivation of embryonic lung progenitor-like transcriptional states in HNRNP-mutant tumors. Genes associated with epithelial organization and cell motility were also enriched, including ENAH and CDC42BPA (actin cytoskeleton dynamics and migration) and CTNNA3 (adherens junction stability). These alterations indicate enhanced epithelial plasticity and invasive potential, resembling cellular remodeling during embryonic lung-branching morphogenesis. Hence, these findings suggest that HNRNP-associated tumors acquire coordinated developmental and metabolic adaptability.

## 4. Discussion

In this study, we constructed a spatiotemporally resolved atlas of embryonic lung development, enabling us to define discrete cellular transitions and regulatory programs governing epithelial–mesenchymal interactions. To more accurately identify the spatial distribution of cell types, we developed a bin-based spatial cell-type annotation strategy that enabled spatial annotation at single-cell resolution. By leveraging the correspondence between bins and cell bins, our approach substantially improved the stability of deconvolution-based cell-type proportion estimation. By leveraging optimal cell-type matching, our approach provided internal validation of cell identity, thereby further enhancing annotation accuracy. Together, this strategy integrated single-cell and spatial transcriptomic datasets of mouse embryonic lung development from E10.5 to E18.5 and precisely mapped the spatial distribution of mesenchymal cell subtype classifications. A total of five distinct mesenchymal subtypes were identified, including embryonic early-stage mesenchymal fibroblasts (C0), myofibroblasts (C1), proliferative fibroblasts (C2), Mecom^+^ mesenchymal population (C3) and embryonic late-stage fibroblasts (C4). This lineage classification was consistent with the remarkable heterogeneity previously reported in lung mesenchymal cells exhibited by lung mesenchymal cells during developmental processes, and further expanded current understanding of the progressive specification of mesoderm-derived cells throughout their spatiotemporal evolution [30,56].

During lung morphogenesis, both fibroblasts and epithelial cells underwent a concomitant transition from an early stage marked by high proliferation to a late stage characterized by increased plasticity in association with structural and functional maturation. Based on our data, we further summarized a conceptual scheme of genes involved in different processes of lung development, which highlighted stage-specific functional transitions. Specifically, the mesenchymal C0 subtype decreased markedly at E13.5, concomitant with the differentiation of these cells into the highly proliferative C2 subtype and maturation-associated C4 subtype. This late stage was accompanied by the downregulation of genes associated with cell proliferation (*Top2a*, *Hmga2*), progenitor-state maintenance (*Ctnnb1*, *Wnt7b*, *Sox2*, *Hes1*) and embryonic development (*Bmpr1b*, *Gata4*), as well as the upregulation of genes associated with extracellular matrix remodeling (*Col18a1*, *Col6a3*, *Eln*, *Slc36a2*) and terminal differentiation (*Bmp6*, *Fgfr3*, *Zbtb16*, *Scube2*). Within this scheme, early developmental processes were primarily associated with proliferative expansion and progenitor maintenance, whereas late-stage processes reflected matrix organization and tissue stabilization. These transcriptional changes corresponded to the previously described reconfiguration of the proximal–distal gene expression pattern in the lung airway as described in other spatial transcriptomic studies [57]. Early epithelial cells displayed gene expression signatures associated with cell migration (*Bmp7*, *Fbln1*, *Mdk*, *Thbs1*), junction remodeling (*Fscn1*, *Fn1*, *Tenm3*, *Cldn6*) and tubular morphogenesis (*Adamts19*, *Lgr5*, *Fn1*, *Hhip*, *Lama1*), whereas late-stage epithelial cells were enriched for programs tightly linked to mature lung function, including surfactant homeostasis (*Ctsh*, *Napsa*, *Sftpb*, *Slc34a2*), endothelial migration (*Ctsh*, *Dapk2*, *Fgf1*, *Lgals3*) and small GTPase signaling (*Aqp1*, *Cd36*, *Cav1*, *Ctsh*, *Lrrk2*). Accordingly, our model suggested that epithelial development progressed from a dynamic remodeling state toward functional specialization, paralleling mesenchymal maturation dynamics. These findings indicated that the epithelial lineage underwent a coordinated transition similar to that of mesenchymal cells and supported an integrated model in which developmental programs are temporally and spatially orchestrated across lineages.

We identified E13.5 as a previously underappreciated transition window during which both mesenchymal and epithelial cells synchronously advanced their developmental trajectories. While earlier studies had described gradual developmental progression, our data revealed a sharp shift in cell-state composition and signaling activity, suggesting that lung development may involve discrete phase transitions rather than continuous changes.

Through analysis of incoming and outgoing signaling activities between the embryonic and pseudoglandular stages, we observed marked upregulation of multiple mesenchymal signaling pathways during the pseudoglandular stage, including BMP, Slit, and NEGR signaling, as well as collagen- and laminin-associated interactions (Figure 4A–D). CellChat analyses further revealed pathway-specific epithelial–mesenchymal communication modules, such as BMP signaling, which was predominantly driven by fibroblasts and mediated through Bmp5–BMPR interactions with epithelial lineages, supporting a central role for BMP signaling in coordinating epithelial differentiation and mesenchymal maturation [43,58]. Meanwhile, we also found that epithelial-derived signaling pathways, including THBS, Nectin, FGF, NRG and CSPG4, exhibited enhanced activity during the pseudoglandular stage. These signals were tightly associated with basement membrane stabilization, establishment of epithelial polarity, and cell–cell as well as cell–ECM adhesion, which indicates that during the pseudoglandular stage, epithelial cells gradually transitioned from early developmental programs dominated by ECM deposition and cell migration to a mature state linked to tissue homeostasis and structural stability. Interestingly, FGF exhibited strong mesenchymal-to-epithelial directionality, primarily mediated by Fgf10-Fgfr2 interactions [59], while PDGF signaling was more spatially restricted, characterized by epithelial-derived ligands acting on mesenchymal Pdgfra-expressing fibroblasts [60,61]. Together, these signaling programs contributed to proximal–distal patterning and structural maturation during the pseudoglandular stage, consistent with the established role of BMP regulatory networks in lung-branching morphogenesis.

To further connect upstream signaling pathways with downstream biological processes, we applied the NicheNet approach to infer the regulatory relationships between ligands and TFs. Among these, we identified a key regulatory axis involving the Hedgehog ligand Shh and its downstream effector Gli1. Previous studies demonstrated that *Shh* serves as a critical regulatory signal for lung-branching morphogenesis and mesenchymal proliferation, and its ablation led to abnormal bronchopulmonary structural development [60]. The epithelial-derived *Shh* ligand could activate *Gli1* signaling in mesenchymal cells during lung development.

Additionally, in mesenchymal cells, we identified two additional TF regulatory modules with distinct temporal activity patterns besides Gli1(+): Tbx3(+) and Tbx5(+). Tbx3(+) regulon exhibited relatively high activity at early developmental stages, and its target genes included multiple components in the SHH, PDGF and BMP pathways (e.g., *Ptch1*, *Pdgfra*, *Smad5*). Tbx5(+) regulon exhibited enhanced regulatory activity during the pseudoglandular stage, and its regulatory targets encompassed Tbx family genes, *Meis1*, *Zeb2*, and a series of Rho GTPase-related genes, reflecting enhanced regulation of cytoskeletal remodeling and directed cell migration. This observation was consistent with the established interaction model of *Tbx5* and *Shh* in coordinated cardiopulmonary development [50], indicating that Tbx5 contributed to lung tissue patterning through coordinated regulation of multiple signaling pathways [61].

Next, we integrated spatial transcriptomic data with lung cancer-associated GWAS data to assess associations between developmental cell states and lung cancer genetic risk. Genes associated with lung cancer risk exhibited progressive downregulation across epithelial cell lineages, which was consistent with the concept that cancer cells reverted to proliferative and plastic cellular states characteristic of early developmental stages, rather than maintaining mature cellular homeostasis [62,63].

By integrating developmental lung signatures with tumor genomic alterations, we found that HNRNP family-associated mutations defined a distinct molecular subgroup characterized by developmental reactivation, cytoskeletal remodeling, and metabolic adaptation. One of the most important observations in this study was the strong enrichment of RNA-binding proteins and spliceosome-associated factors within the embryonic lung-associated gene signature. HNRNP family proteins are essential regulators of RNA maturation, alternative splicing, and transcript stability during development. During embryonic lung morphogenesis, dynamic RNA processing is required for progenitor cell specification, epithelial branching, and lineage differentiation. Our findings suggest that LUAD tumors may hijack these developmental RNA regulatory mechanisms to promote malignant progression. Our study extends the developmental context of HNRNP dysregulation by demonstrating that HNRNP alterations are specifically associated with embryonic lung progenitor-related transcriptional programs in TP53-mutant LUAD.

## 5. Conclusions

In summary, we constructed a spatiotemporally resolved framework of embryonic lung development and identified E13.5 as a critical developmental transition window coordinating epithelial and mesenchymal lineage specialization. Our findings revealed dynamic regulatory programs and intercellular signaling networks underlying bronchial and alveolar morphogenesis, providing mechanistic insights into lung developmental patterning. Furthermore, this study established a developmental framework for understanding the molecular basis of lung diseases and offered potential implications for regenerative medicine and therapeutic intervention strategies.

## Data Availability

Spatial transcriptome data downloaded from: https://db.cngb.org/stomics/mosta/ (accessed on 25 November 2025). Single-cell data downloaded from: https://omg.gs.washington.edu/ (accessed on 1 December 2025).

## Supplementary Materials

The following supporting information can be downloaded at: https://www.mdpi.com/article/10.3390/jdb14020025/s1, Figure S1: Spatiotemporal and Single-Cell Data Quality Control and Signature Gene Visualization; Figure S2: Pipeline of the bin-based deconvolution method and lung tissue annotation outcomes; Figure S3: Identification of early and late epithelial signatures and the transition window of epithelial and mesenchymal states; Figure S4: GO enrichment of transcription factor activity modules; Figure S5: Spatiotemporal Dynamics of Epithelial and Mesenchymal Signatures; Table S1: Marker genes of mesenchymal cells in the early and late stages; Table S2: Marker genes of epithelial cells in the early and late stages.

## Abbreviations

ECM	extracellular matrix
PCA	principal component analysis
IGF	insulin-like growth factor
AT1	alveolar type I
AT2	alveolar type II
ADGRL	Adhesion G Protein-Coupled Receptor L
NEGR	Neuronal Growth Regulator
BMP	Bone Morphogenetic Protein
Pcdh	Protocadherin
CADM	Cell Adhesion Molecule
PDGF	Platelet-Derived Growth Factor
CDH	Cadherin
FGF	Fibroblast Growth Factor
RA	Retinoic acid
SHH	Sonic Hedgehog
GO	Gene Ontology
