# Supplementary figures and images for: Spatiotemporal Profiling Defines the Epithelial and Mesenchymal Transition Window in Embryonic Lung Morphogenesis
Source: J Dev Biol. 2026 Jun 1;14(2):25. doi: 10.3390/jdb14020025 (PMC13300856; doi:10.3390/jdb14020025)

Supplementary Figure S1

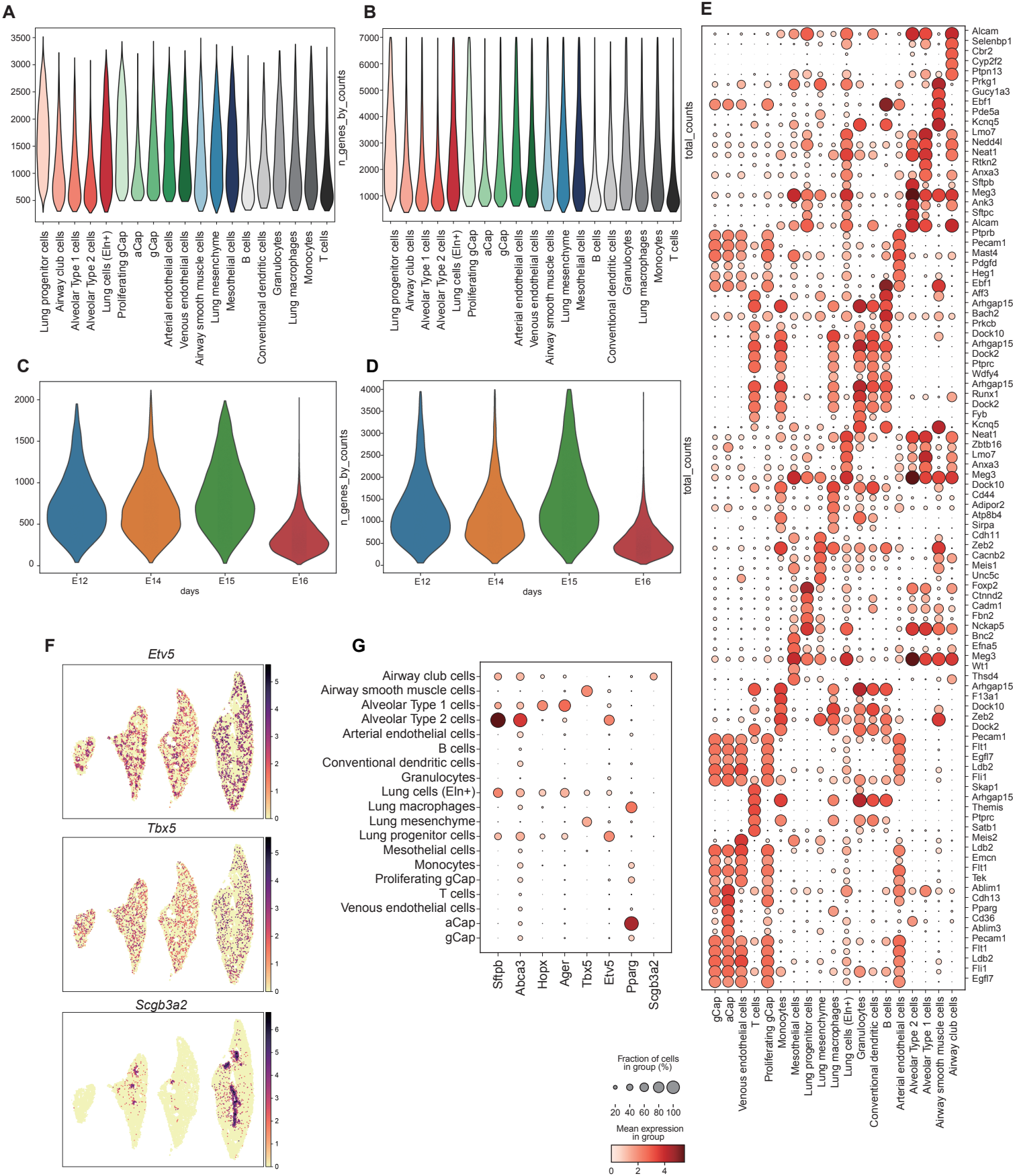

Supplement: Supplementary file 1 [file jdb-14-00025-s001.zip › jdb-4176846-supplementary/Supplementary Figure S1.pdf]

Supplementary Figure S2

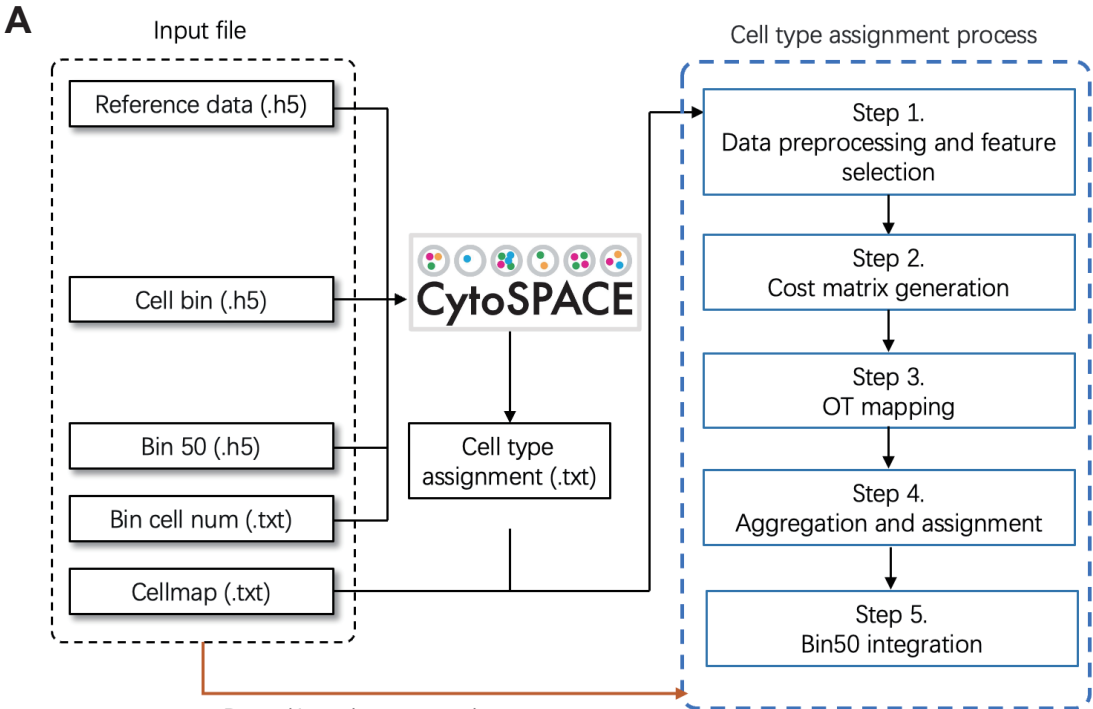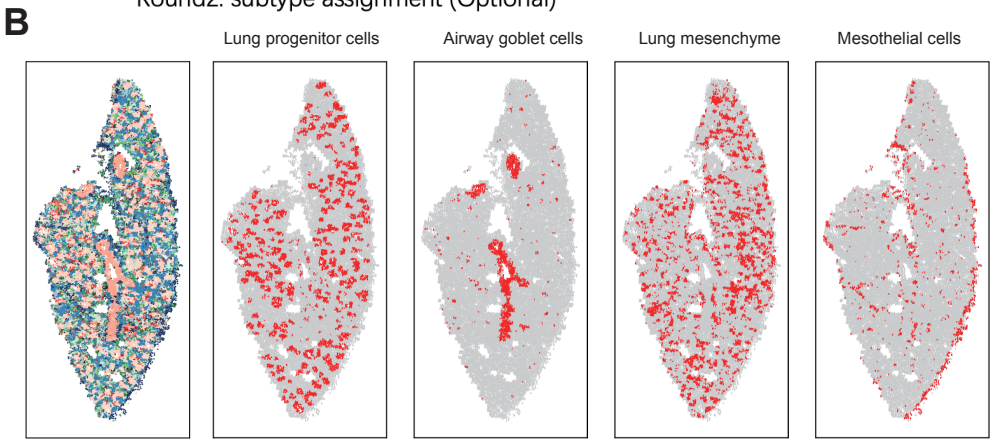

The results of the CytoSpace single-cell annotation process

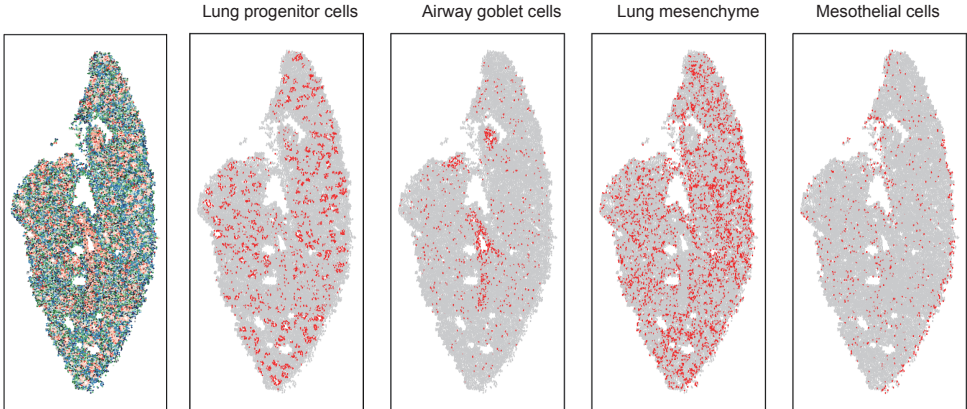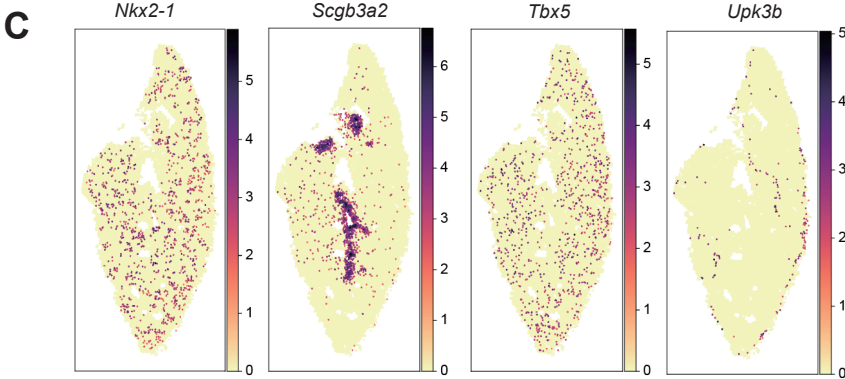

Supplement: Supplementary file 1 [file jdb-14-00025-s001.zip › jdb-4176846-supplementary/Supplementary Figure S2.pdf]

# Supplementary Figure S3

A

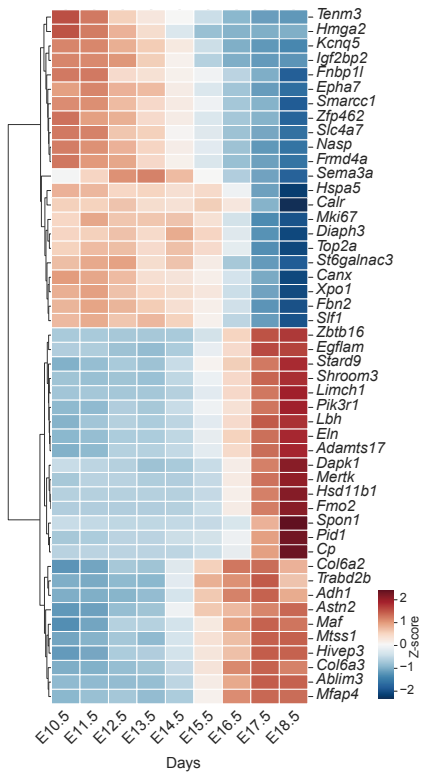

B

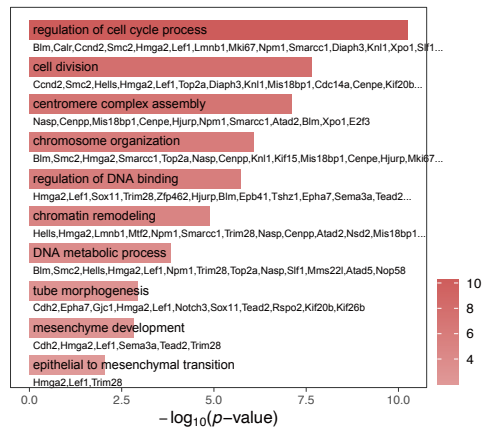

C

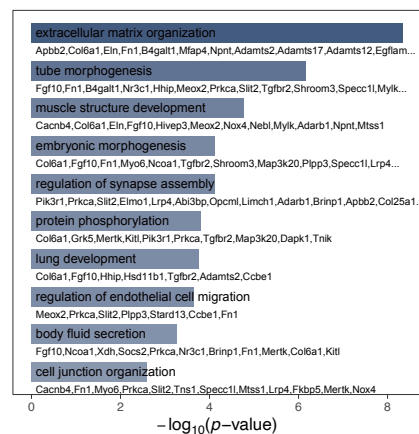

D

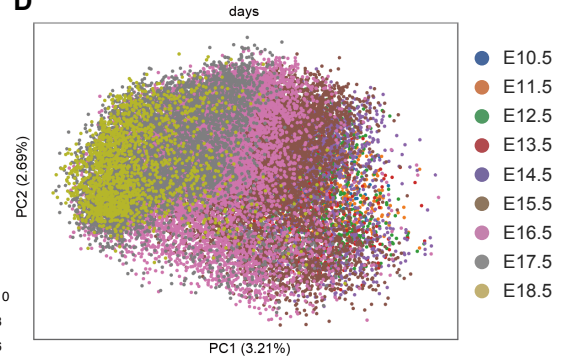

E

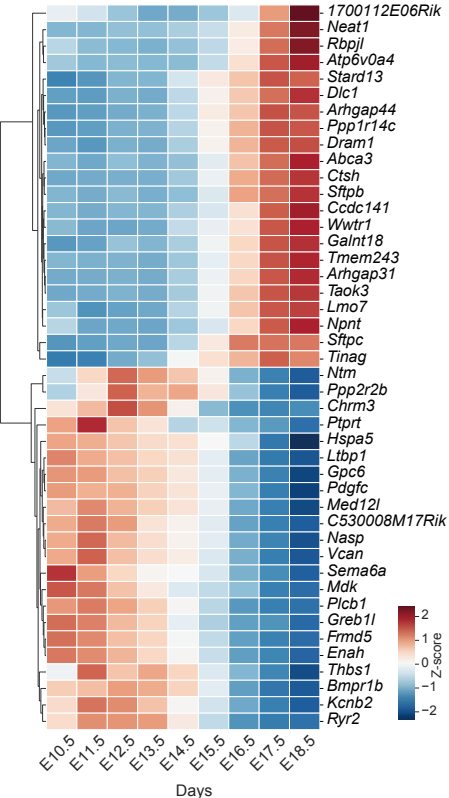

F

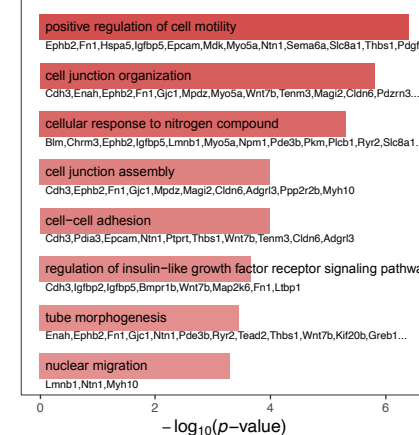

G

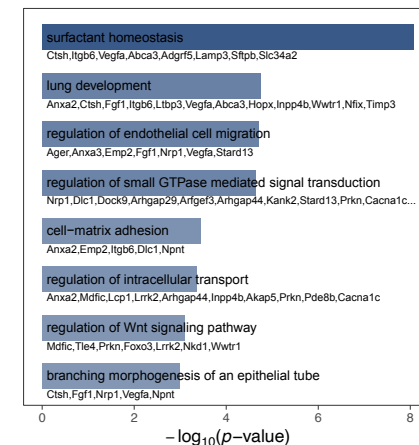

Supplement: Supplementary file 1 [file jdb-14-00025-s001.zip › jdb-4176846-supplementary/Supplementary Figure S3.pdf]
