# Supplementary material for: Spatiotemporal Profiling Defines the Epithelial and Mesenchymal Transition Window in Embryonic Lung Morphogenesis
Source: J Dev Biol. 2026 Jun 1;14(2):25. doi: 10.3390/jdb14020025 (PMC13300856; doi:10.3390/jdb14020025)

Supplementary Figure S4

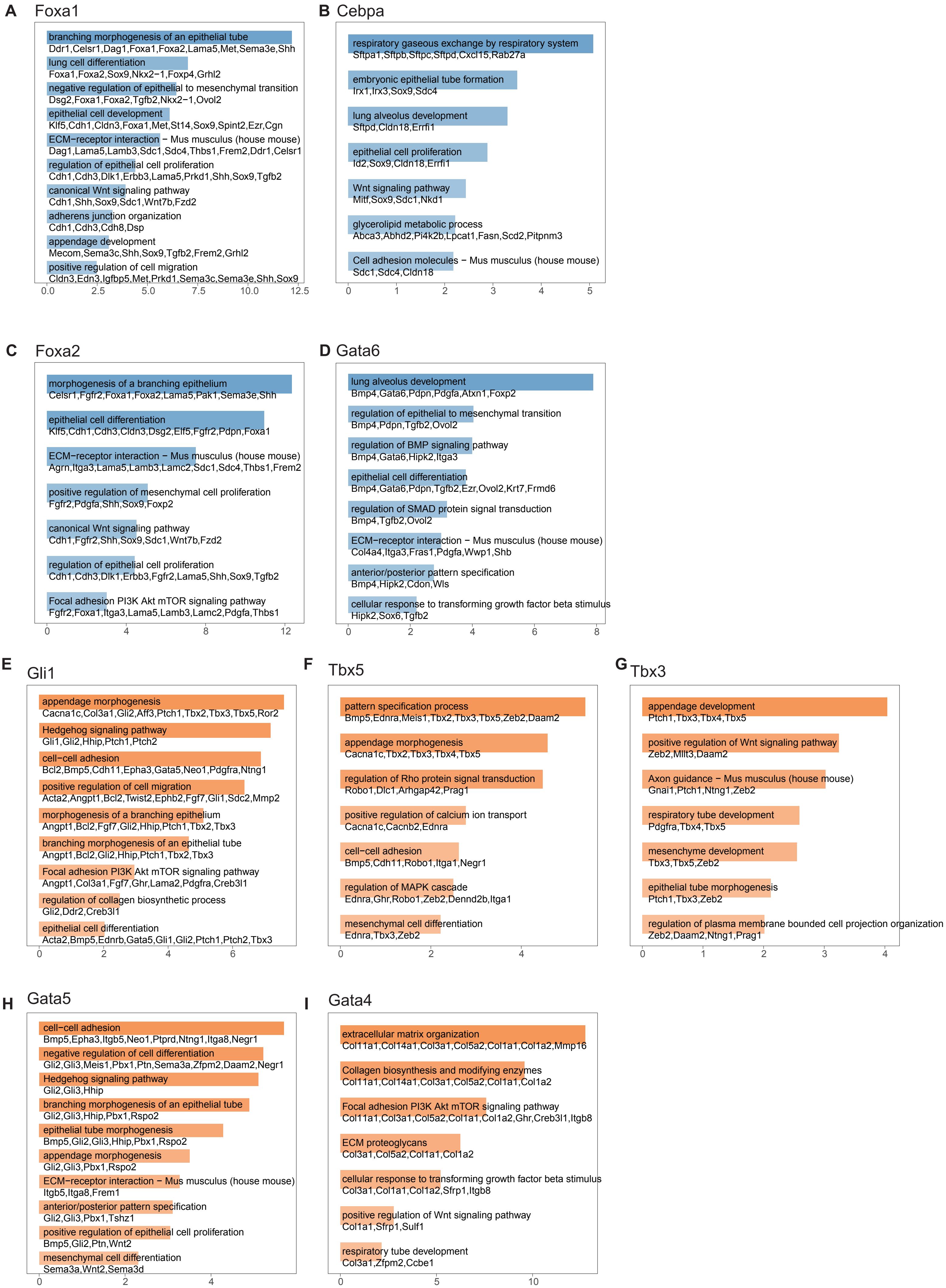

Supplement: Supplementary file 1 [file Supplementary Figure S4.pdf › jdb-4176846-supplementary/Supplementary Figure S4.pdf]
